# Targeting the hemangioblast with a novel cell type-specific enhancer

**DOI:** 10.1186/1471-213X-11-76

**Published:** 2011-12-28

**Authors:** Vera Teixeira, Natacha Arede, Rui Gardner, Joaquín Rodríguez-León, Ana T Tavares

**Affiliations:** 1 Instituto Gulbenkian de Ciência, 2780-156 Oeiras, Portugal; 2 Facultad de Medicina, Universidad de Extremadura, 06006 Badajoz, Spain; 3 Centro de Investigação Interdisciplinar em Sanidade Animal (CIISA), Faculdade de Medicina Veterinária, Universidade Técnica de Lisboa, 1300-477 Lisboa, Portugal

## Abstract

**Background:**

Hemangioblasts are known as the common precursors for primitive hematopoietic and endothelial lineages. Their existence has been supported mainly by the observation that both cell types develop in close proximity and by in vitro differentiation and genetic studies. However, more compelling evidence will arise from tracking their cell fates using a lineage-specific marker.

**Results:**

We report the identification of a hemangioblast-specific enhancer (Hb) located in the *cis*-regulatory region of chick *Cerberus *gene (*cCer*) that is able to direct the expression of enhanced green fluorescent protein (eGFP) to the precursors of yolk sac blood and endothelial cells in electroporated chick embryos. Moreover, we present the Hb-eGFP reporter as a powerful live imaging tool for visualizing hemangioblast cell fate and blood island morphogenesis.

**Conclusions:**

We hereby introduce the Hb enhancer as a valuable resource for genetically targeting the hemangioblast population as well as for studying the dynamics of vascular and blood cell development.

## Background

In the early vertebrate embryo, both hematopoietic and endothelial lineages derive from aggregates of mesodermal cells that form the blood islands in the extraembryonic yolk sac [1]. This observation led to the hypothesis that both lineages derive from a common precursor named the hemangioblast [2]. Although still debatable, the existence of hemangioblasts is mainly supported by *in vitro *differentiation studies [3,4] as well as by evidence that blood and endothelial progenitors express a number of genes in common, such as *VEGFR2*, *GATA2*, *Lmo2 *and *Scl/Tal1 *[5], some of which regulate the differentiation of both cell lineages [6-8]. Other than these in vitro and genetic studies, further insight into hemangioblast cell fate will require time-lapse imaging studies using a lineage-specific marker.

Hemangioblast reporters have been described in transgenic mouse and zebrafish models [9,10]. However, a particularly suitable system for visualizing live hematovascular development is the yolk sac of the avian embryo [11,12]. During the study of chick *Cerberus *(*cCer*) transcriptional regulation [13], we isolated a *cis*-regulatory region that drives reporter gene expression specifically in blood-island progenitors or hemangioblasts. Here, we report the identification and characterization of this novel hemangioblast-specific enhancer and reveal its potential as a live imaging tool for studying blood and vascular development.

## Results and Discussion

**Figure 1:**
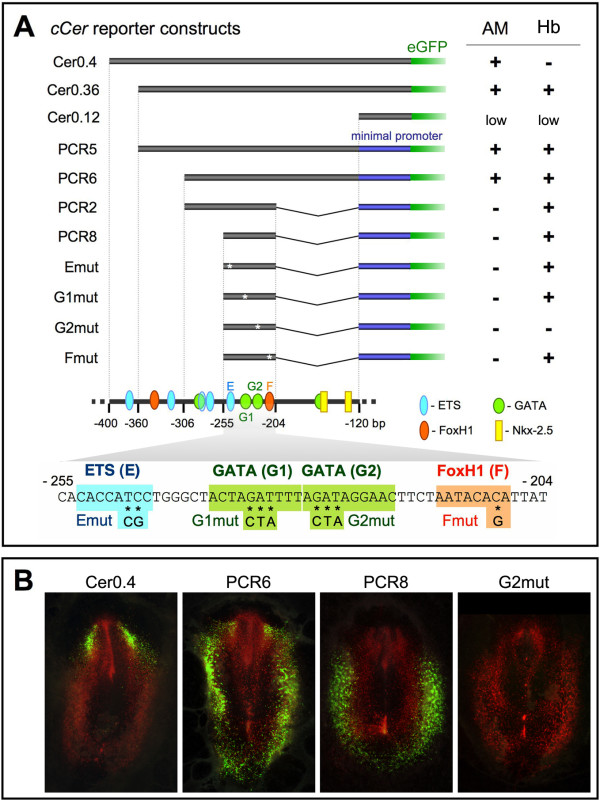
**Identification of the *cCer *hemangioblast enhancer**. (**A**) Enhancer analysis of the *cCer cis*-regulatory region. *cCer *5' genomic sequences (black boxes) were either directly fused to the eGFP reporter gene (green boxes) or sub-cloned into an enhancerless vector carrying the human β-globin minimal promoter (dark blue boxes) upstream of the eGFP coding sequence. Emut, G1mut, G2mut and Smut constructs were designed by introducing mutations in the ETS, GATA (G1 and G2) or FoxH1 (F) binding elements of the PCR8 sequence, respectively (asterisks; see sequence below). The presence ("+") or absence ("-") of eGFP expression in the anterior mesendoderm (AM) and in hemangioblasts (Hb) of electroporated chick embryos is listed on the right. Each result is representative of at least 12 embryos. A schematic representation of *cCer *-400 to -120 bp regulatory region and the nucleotide sequence of the PCR8 fragment (-255 to -204 bp) are shown in the bottom. Binding sites for the transcription factors ETS (E; blue), GATA (G1 and G2; green), FoxH1 (F; orange) and Nkx-2.5 (yellow) are outlined. The ETS site in the -400 to -360 bp silencing region may be responsible for the repression of hemangioblast expression, whereas the two Nkx-2.5 sites in the -204 to -120 bp sequence may regulate anterior mesendoderm expression. Mutations introduced into the E, G1, G2 and F sites of the Emut, G1mut, G2mut and Smut constructs are also indicated in the PCR8 sequence. (**B**) Cer-eGFP reporter expression in electroporated chick embryos. Embryos were co-transfected with pCAGGS-RFP (positive control; red fluorescence) and each Cer-eGFP reporter construct (green fluorescence) at stage HH3 and fixed at HH6. Examples of electroporated embryos with ubiquitous RFP fluorescence and specific eGFP expression in the AM (Cer0.4), AM and Hb (PCR6), and Hb alone (PCR8), or without eGFP expression (G2mut).

**Figure 2:**
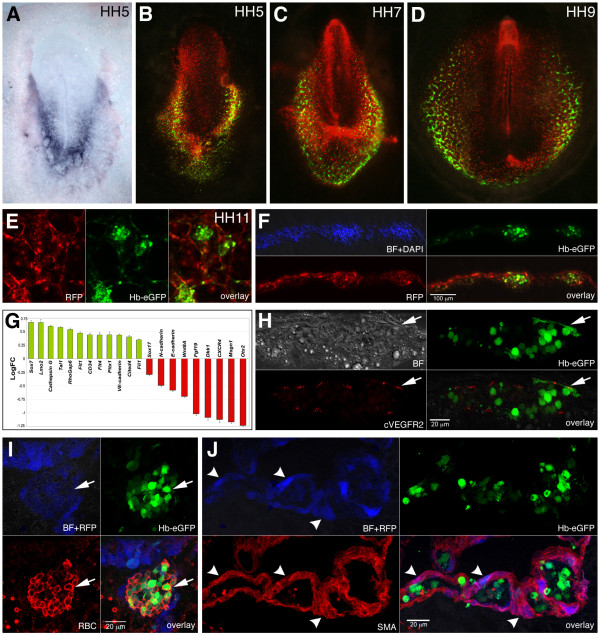
**Characterization of the Hb-eGFP-positive cell population**. Chick embryos were processed for cVEGFR2 whole mount in situ hybridization at HH5 (A) or co-electroporated at HH3-4 with the Hb-eGFP reporter construct (PCR2) and the ubiquitous reporter pCAGGS-RFP (B-J) and imaged as whole mounts at HH5 (B), HH7 (C), HH9 (D) and HH11 (E), criosectioned through the yolk sac region at HH11 (F, H-J), or processed for microarray analysis at HH5-6 (G). (B-D) Overlay of bright field, Hb-eGFP green fluorescence and RFP red fluorescence images. (E) RFP fluorescence (left), Hb-eGFP fluorescence (middle) and overlay (right). (F, H-J) Top left: bright field and DAPI (F), bright field alone (H), or bright field and RFP (in blue; I and J); top right: Hb-eGFP green fluorescence; bottom left: RFP red fluorescence (F) or immunolabeling of cVEGFR2 (H), red blood cells (RBC; I) and smooth muscle actin (SMA; J); bottom right: overlay of bright field and fluorescence images. At HH5, cVEGFR2 expression is detected in the posterior extraembryonic population of hemangioblasts (**A**). At this early stage, Hb-eGFP fluorescence is specifically observed in the same cell population (**B**). At later stages, eGFP-fluorescent cells aggregate in the extraembryonic region (**C**), give rise to blood islands (**D**), and integrate the vascular plexus of the yolk sac (**E**). Note that the control RFP reporter is ubiquitously expressed, whereas Hb-eGFP fluorescence is restricted to the blood islands (E and F). At early stages, the gene expression profile of Hb-eGFP-positive cells revealed that known hemangioblast markers are enriched, whereas genes expressed in other tissues are downregulated in this cell population (**G**; see also Additional file 2). At later stages, Hb-eGFP fluorescence is detected in cVEGFR2-positive endothelial cells (**H**; arrow) and blood cells or erythroblasts (**I**; arrows), but not in smooth muscle cells (**J**; arrowheads).

To study the transcriptional activity of *cCer cis*-regulatory region, chick embryos were electroporated with reporter constructs containing *cCer *5' genomic sequences of different lengths upstream of the coding sequence for enhanced green fluorescent protein (eGFP), and observed under a fluorescent microscope (Figures 1 and 2) [13]. Our initial results showed that a 400-base pairs (bp) promoter fragment upstream from the ATG of *cCer *(Cer0.4) is able to drive eGFP expression in cell populations that express *cCer*, i.e., the anterior mesendoderm (Figure 1) and the left-side mesoderm [13,14]. Further deletion analysis revealed an ectopic domain of expression in the posterior extraembryonic mesoderm where hemangioblasts are located [5]. This pattern was observed in embryos electroporated with constructs that lack the -400 to -360 bp sequence (*i.e*., Cer0.36, PCR5 and PCR6; Figure 1), suggesting that this region may contain a silencer of hemangioblast expression. Cer-eGFP expression was abolished in the anterior mesendoderm and restricted to hemangioblasts in embryos electroporated with constructs that lack the -204 to -120 bp sequence (*i.e*., PCR2 and PCR8; Figure 1 and data not shown). PCR8 regulatory sequence is hereafter named the hemangioblast or Hb enhancer, and the hemangioblast-specific reporters PCR2-eGFP and PCR8-eGFP are named Hb-eGFP.

Sequence analysis of the Hb enhancer identified binding sites for transcription factors implicated in endothelial and blood cell differentiation, namely ETS, GATA and FoxH1 [15-17] (Figure 1A). To determine which of these binding elements may be responsible for the regulation of hemangioblast-specific expression, we analyzed the expression of reporter constructs containing mutations in the ETS (E), GATA (G1 and G2) or FoxH1 (F) sites in the PCR8 sequence [17-19] (Figure 1A). Hemangioblast expression was unaltered in embryos electroporated with the Emut, G1mut and Fmut constructs, but abolished in those electroporated with the G2mut construct (Figure 1B). These observations demonstrate that the G2 site is essential for the induction or maintenance of transcription in hemangioblasts, and suggest that the GATA2 is a transcriptional activator of the Hb enhancer in blood-island progenitors [5].

In the avian embryo, hemangioblasts ingress through the posterior primitive streak between stages HH2 and HH9 [20]. To characterize the expression pattern of the Hb-eGFP reporter, embryos were electroporated at either early or late stages (HH3-4 and HH5-6, respectively), placed in culture and observed at successive time points (HH4-13). In embryos electroporated at HH3-4, Hb-eGFP fluorescence was initially detected in posterior primitive streak cells (data not shown) and in a cVEGFR2-positive population of posterior extraembryonic cells (HH5; Figures 2A and 2B). At later stages, eGFP-positive cells migrate away from the embryo and form aggregates in the extraembryonic region (HH7; Figure 2C) that will give rise to the blood islands (HH9; Figure 2D). At HH11, Hb-eGFP fluorescence was restricted to differentiated blood islands and the vascular cells that connect them (Figure 2E and 2F). In embryos electroporated at HH5-6, Hb-eGFP expression was specifically detected in the blood islands that form closer to the embryo (Additional file 1). Taken together, these observations suggest that the Hb enhancer is continually activated in ingressing hemangioblasts that populate the extraembryonic region in a lateral to medial temporal progression [21].

To confirm the specificity of the Hb-eGFP reporter, we analyzed the gene expression profile of early Hb-eGFP-positive cells and investigated the co-localization of eGFP fluorescence with known markers of hemangioblast-derived cells. As expected, genes expressed in hemangioblasts, such as *Lmo2 *(+3.73 fold), *Tal1 *(+3.62 fold) and *CD34 *(+2.39 fold), are enriched in the Hb-eGFP-positive population, whereas those expressed in other cell types, such as the endoderm (e.g., *Sox17*), neuroectoderm (e.g., *Otx2*) and paraxial mesoderm (e.g., *Msgn1*), are downregulated (Figure 2G; Additional file 2). At a later stage (HH11), Hb-eGFP fluorescence is detected both in endothelial cells, which express cVEGFR2 protein (Figure 2H), and in blood cells, which express the RBC antigen (Figure 2I). However, Hb-eGFP expression is not observed in the SMA-positive smooth muscle cells that surround the blood islands (Figure 2J). This observation supports the hypothesis that smooth muscle cells are not derived from hemangioblasts [22].

Taken together, our observations indicate that Hb-eGFP expression is initiated in hemangioblasts as they emerge from the primitive streak and is detected in differentiated endothelial and blood cells at least until HH13 (data not shown). This pattern was confirmed by time-lapse imaging of live electroporated embryos. At low amplification, we could see the eGFP-fluorescent cells moving away from the posterior primitive streak, aggregating to form the blood islands, and giving rise to the vascular plexus by connecting the separate islands (Additional file 3). At higher magnification, we were able to follow the movements of individual endothelial and blood cells (Additional file 4), such as the interchange of cells between different blood islands and the cell division of an erythroblast. Moreover, in older embryos, the Hb-eGFP reporter proved to be a very useful tool to record blood cell flow in the vascular plexus of the yolk sac (Additional file 5).

## Conclusions

In summary, we identified a hemangioblast enhancer located in *cCer cis*-regulatory region and describe its activity in developing chick embryos. Furthermore, we have used the Hb-eGFP reporter to characterize the gene expression profile of hemangioblasts and visualize blood island morphogenesis and differentiation in living embryos. In the future, the Hb-eGFP reporter may become a valuable genetic tool for targeting ectopic gene expression to the hemangioblast population as well as for studying live vasculogenesis and blood flow.

## Methods

### DNA constructs

The isolation and cloning of *cCer *5' genomic sequences was performed as previously reported [13]. In particular, *cCer *regulatory sequences were amplified by PCR using the Cer0.36-eGFP construct DNA as template (primer sequences provided upon request), and sub-cloned into the *Sac*I/*Spe*I restriction sites of the p1229-eGFP enhancerless vector, which carries the human beta-globin minimal promoter [23] upstream of the eGFP coding sequence (Clontech). Putative binding elements for ETS, GATA (sites 1 and 2) and FoxH1 transcription factors were identified in the analysis of *cCer *genomic sequences using MatInspector Professional release 7.4 [24] and MatchTM [25]http://www.gene-regulation.com/. Mutations in these elements were designed according to the literature [18,19,26] and introduced into the PCR8 construct by PCR-based site-directed mutagenesis. The pCAGGS-*RFP *vector (gift from D. Henrique), which contains the CAGGS promoter and the cDNA of monomeric red fluorescent protein (RFP; Clontech) [27] was used to control the electroporation efficiency.

### Embryo electroporation and imaging

Chicken embryos were explanted and electroporated at stages HH3-5 [28] as described previously [13]. Electroporated embryos were grown in New culture [29] until stages HH6-11, observed under a Zeiss SteREO Lumar fluorescence stereomicroscope (Carl Zeiss) and photographed using a Hamamatsu C8484 digital camera (Hamamatsu Photonics) and AxioVision software (Carl Zeiss).

### Fluorescence-activated cell sorting (FACS)

Chicken embryos were electroporated at HH3 with Hb-eGFP and pCAGGS-RFP constructs, harvested at stage HH5-6 into three groups of four embryos each, dissociated into single cell suspensions using trypsin (Sigma-Aldrich) and filtered through a 35 μm cell strainer (BD Bioscience). The eGFP+ and eGFP-/RFP+ cell populations were FACS-sorted in a Moflo high-speed cell sorter (Beckman Coulter), using a 70 μm ceramic nozzle with 0.414MPa (60 psi) sheath pressure, a 488 nm laser line from a Coherent Sapphire 488-200 CDRH laser for eGFP excitation, and a 561 nm laser line from a CrystaLaser GCL-050-561 50 mW DPSS laser coupled to fiber optics (38 mW output) to excite RFP. eGFP+ and RFP+ cells were detected using 530/40 nm and 630/75 nm HQ band pass filters, respectively, and collected simultaneously into two different tubes containing RNAlater (Ambion).

### RNA Isolation and Microarray Expression Analysis

Total RNA was extracted from triplicates of each cell population using the RNeasy Mini Kit (Qiagen). Concentration and purity was determined by spectrophotometry and integrity confirmed using an Agilent 2100 Bioanalyzer with a RNA 6000 Nano Assay (Agilent Technologies). Prior to processing for microarray hybridization, RNA samples from FACS-sorted eGFP+ and eGFP-/RFP+ populations were analyzed for the expression of control and marker genes (*GAPDH*, *eGFP*, *RFP*, *cVEGFR2*, *cLmo2 *and *cBra*) by reverse transcription polymerase chain reaction (data not shown). For each of the six samples, 40 ng of total RNA was processed according to the manufacturer's Two-Cycle Target Labeling Assay. Size distribution of the cRNA was assessed using an Agilent 2100 Bioanalyzer with a RNA 6000 Nano Assay. Affymetrix GeneChip Chicken Genome Arrays were hybridized at 45°C for 16 h with 15 μg of fragmented cRNA, washed and double-stained on an Affymetrix GeneChip Fluidics Station 450, and scanned on an Affymetrix GeneChip scanner 3000 7G. The arrays were analyzed using Affymetrix GCOS 1.4 and dChip 2008 software (http://www.dchip.org, Wong Lab, Harvard). Normalized CEL intensities of the six arrays were used to obtain gene expression indices based on a Perfect Match-only model. Only genes with a lower 90% confidence bound of the fold change above 1.7 were regard as differentially expressed and used for further analysis. Annotations for the 33.457 transcripts that are represented on the GeneChip Chicken Genome Array were obtained from the NetAffx database http://www.affymetrix.com as of September 2008. Out of this total number of transcripts, 658 were differentially expressed in the two populations, including 476 known genes (fold change > 1.7). Of these, 170 were upregulated and 306 downregulated in Hb-eGFP+ cells. Microarray data have been deposited in NCBI's Gene Expression Omnibus (GEO) with the accession number GSE32494.

### In situ hybridization and Immunohistochemistry

Whole-mount in situ hybridization was performed as described previously [14]. The cVegfr2 riboprobe fragment (nucleotides 2488-2771) was generated by RT-PCR and cloned into pGEMTeasy vector (Promega). Embryo images were acquired using a Leica MZ FLIII stereomicroscope, a Leica DFC420C digital camera (Leica Microsystems) and IrfanView software http://www.irfanview.com/.

For immunohistochemistry, electroporated embryos were fixed in 4% paraformaldehyde, cryoprotected in 15% sucrose, embedded in 7.5% gelatine/15% sucrose and cryosectioned at 16 or 20 μm. Immunostaining was performed using primary antibodies against avian VEGFR2 (gift from Anne Eichmann) [3], chicken Red Blood Cells (RBC; #103-4139; Rockland Immunochemicals), and alpha smooth muscle actin (SMA; #ab5694; Abcam), and secondary antibodies labeled with the fluorescent probe Alexa Fluor 568 (#A11004; Molecular Probes/Invitrogen) or Alexa Fluor 647 (#A21245; Molecular Probes). Cell nuclei were labeled with 4', 6-diamidino-2-phenylindole (DAPI; Molecular Probes). Sections were mounted in Fluorescence Mounting Medium (Dako), photographed using either a Leica DMRA2 fluorescence microscope with a HC PL Fluotar 20/0.50 objective (Leica Microsystems), Photometrics CoolSNAP HQ camera (Photometrics) and MetaMorph software (Molecular Devices; Figures 2F, Additional file 1B and 1C), or a Leica TCS SP5 confocal microscope with a HCX PL Apo CS 40/1.40-0.60 objective and Leica Application Suite software (Leica Microsystems; Figures 2H-J). Images were processed and assembled using Photoshop CS3 (Adobe Systems) and Imaris (Bitplane).

## Authors' contributions

VT, NA and ATT carried out experiments and analyzed data. RG performed the FACS assays. JR-L analyzed data and critically commented on the manuscript. ATT designed the study and prepared the manuscript. All authors read and approved the final manuscript.

## Supplementary Material

Additional file 1 **Hb-eGFP expression in chick embryos electroporated at late stages**. Chick embryos were co-electroporated with Hb-eGFP (PCR2) and pCAGGS-RFP reporter constructs at HH5 and fixed at HH11. Top left: bright field (BF); top right: Hb-eGFP green fluorescence; bottom left: RFP red fluorescence; bottom right: overlay of bright field and fluorescence images. Hb-eGFP expression is detected in blood islands (arrows) and in the vascular plexus of the area pellucida (arrowhead). Click here for file

Additional file 2 **List of selected genes up- and down-regulated in Hb-eGFP+ cells at HH5-6**. Listed genes exhibit greater than 1.7-fold change (lower bound) in expression in Hb-eGFP+ versus Hb-eGFP- cells. Gene function and expression patterns are given when known and were obtained from GEISHA http://geisha.arizona.edu/geisha and from the literature. Click here for file

Additional file 3 **Time-lapse movie of a developing chick embryo co-electroporated with Hb-eGFP (PCR2) and pCAGGS-RFP reporter constructs (stages HH4 to HH11)**. As the embryo elongates, Hb-eGFP-positive cells move away from the primitive streak, aggregate to form the blood islands, and give rise to the vascular plexus by connecting the separate islands. Anterior side of the embryo is to the top; images were taken under a Leica DMIRE2 inverted microscope (2.5 × objective; green, Hb-e GFP; red, RFP); 6 min per frame; total time = 24 hours and 12 min; time is indicated in hours in the upper left corner of the image. Click here for file

Additional file 4 **Time-lapse movie of yolk sac blood islands**. The chick embryo was co-electroporated with Hb-eGFP (PCR2) and pCAGGS-RFP reporter constructs at stage HH4 and imaged from HH10 to HH11. eGFP-positive cells are found in blood islands as well as in the vascular-like structures that connect individual blood islands. In this video, we could identify individual cells moving between different blood islands (white arrowhead) as well as the division of a hematopoietic cell in one of the blood islands (yellow arrowheads). Images were taken under a Leica Sp5 confocal microscope (10 × objective; green, Hb-eGFP; red, RFP; small square = 50 mm); 3 min per frame; total time = 4 hours and 6 min; time is indicated in hours in the upper left corner of the image. Click here for file

Additional file 5 **Time-lapse movie of blood cell flow in the yolk sac**. The chick embryo was co-electroporated with Hb-eGFP (PCR8) and pCAGGS-RFP reporter constructs at stage HH4- and imaged at HH12. At this stage, movements of eGFP fluorescent blood cells are already observed within the vascular plexus of the yolk sac. Images were taken under a Leica Sp5 confocal microscope (10 × objective; green, Hb-e GFP; red, RFP); 3 min per frame; total time = 75 min; time is indicated in minutes in the upper left corner of the image. Click here for file
