# Supplementary figures and images for: Targeting the hemangioblast with a novel cell type-specific enhancer
Source: BMC Dev Biol. 2011 Dec 28;11:76. doi: 10.1186/1471-213X-11-76 (PMC3273444; doi:10.1186/1471-213X-11-76)

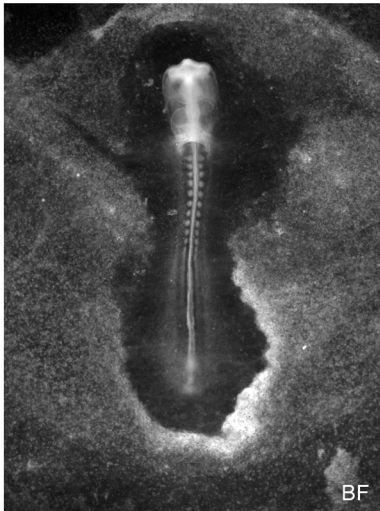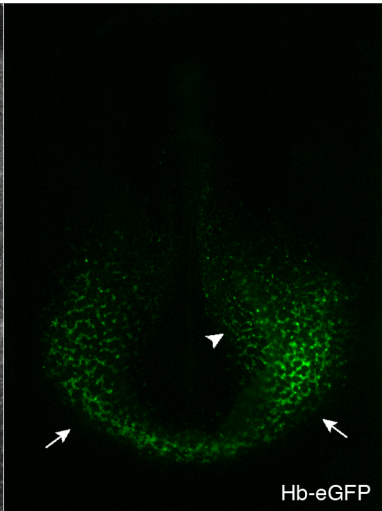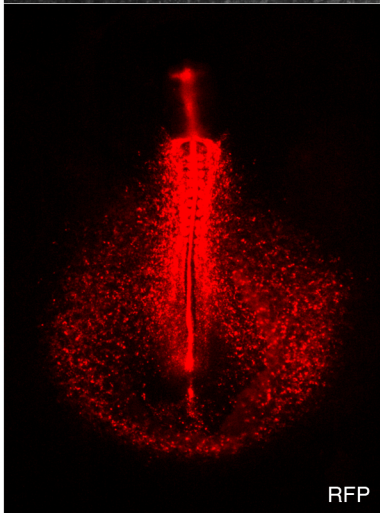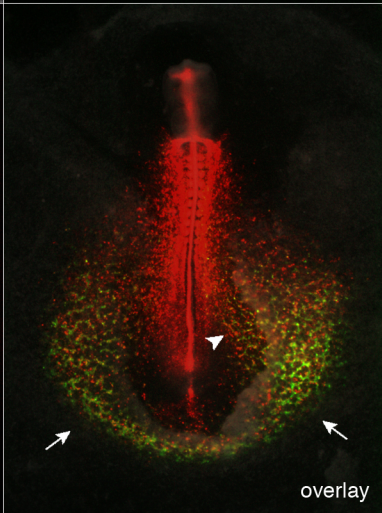

Supplement: Additional file 1 — Hb-eGFP expression in chick embryos electroporated at late stages. Chick embryos were co-electroporated with Hb-eGFP (PCR2) and pCAGGS-RFP reporter constructs at HH5 and fixed at HH11. Top left: bright field (BF); top right: Hb-eGFP green fluorescence; bottom left: RFP red fluorescence; bottom right: overlay of bright field and fluorescence images. Hb-eGFP expression is detected in blood islands (arrows) and in the vascular plexus of the area pellucida (arrowhead). [file 1471-213X-11-76-S1.PDF]
