# Supplementary material for: Targeting the hemangioblast with a novel cell type-specific enhancer
Source: BMC Dev Biol. 2011 Dec 28;11:76. doi: 10.1186/1471-213X-11-76 (PMC3273444; doi:10.1186/1471-213X-11-76)
Supplement: Additional file 2 — List of selected genes up- and down-regulated in Hb-eGFP+ cells at HH5-6. Listed genes exhibit greater than 1.7-fold change (lower bound) in expression in Hb-eGFP+ versus Hb-eGFP- cells. Gene function and expression patterns are given when known and were obtained from GEISHA http://geisha.arizona.edu/geisha and from the literature. [file 1471-213X-11-76-S2.PDF]

Additional file 2

Table 1. List of selected genes up- and down-regulated in Hb-eGFP+ cells at HH5-6.

Fold change	Gene symbol	Gene name	Entrez Gene ID	Molecular function	Biological function	Expression in early embryos
**+15.77**	U43284-1	Green Fluorescent Protein (GFP) sequence	-	Fluorescent marker	-	-
**+5.1**	SLC15A1	Solute carrier family 15, member 1	378789	Membrane transporter	Intestinal peptide transport	-
**+4.2**	LOC418555	Similar to RIKEN cDNA 4930578C19	418555	-	-	-
**+4.15**	SOX7	SRY (sex determining region Y)-box 7	771337	Transcription factor	Vasculogenesis	Endothelial cell precursors
**+4.1**	ChEST995n23	Finished cDNA, clone ChEST995n23	-	-	-	-
**+3.73**	LMO2	LIM domain only 2	374129	Transcription factor	Hematopoiesis	Blood islands and their precursors
**+3.7**	CTSG	Cathepsin G	426049	Serine protease	Tissue remodeling	Early myeloid precursors
**+3.62**	TAL1 (SCL)	T-cell acute lymphocytic leukemia 1 (stem cell leukenia)	396298	Transcription factor	Hematopoiesis	Blood islands and their precursors
**+3.08**	HSP25	Heat shock protein 25	428310	Molecular chaperone	Stress response	Developing blood vessels and heart
**+3.07**	RhoGap 6	Similar to Rho-GTPase-activating protein 6 (LOC422284 locus)	422284	Cytoskeleton regulator	Cell migration	-
**+2.71**	FLT1 (VEGFR1)	Fms-related tyrosine kinase 1 (vascular endothelial growth factor receptor 1)	374100	Receptor tyrosine kinase	Vasculogenesis / Angiogenesis	Blood islands
**+2.63**	SOX18	SRY (sex determining region Y)-box 18	374200	Transcription factor	Vasculogenesis	Blood islands
**+2.49**	CDH5	Cadherin 5, type 2, VE-cadherin (vascular epithelium)	374068	Cell adhesion molecule	Vasculogenesis / Angiogenesis	Endothelial cells
**+2.39**	CD34	Hematopoietic progenitor cell antigen CD34	419856	Cell surface antigen	-	-
**+2.29**	FLT4 (VEGFR3)	Fms-related tyrosine kinase 4 (vascular endothelial growth factor receptor 3)	395742	Receptor tyrosine kinase	Angiogenesis	Blood islands and endothelial cells
**+2.23**	CITED4	Cbp/p300-interacting transactivator, with Glu/Asp-rich carboxy-terminal domain, 4	395465	Transcription regulator	-	Blood islands
**+2.2**	PITX1	Paired-like homeodomain 1	374201	Transcription factor	Pituitary and hindlimb development	Posterior extraembronic mesoderm
**+2.09**	Fli1	Friend leukemia virus integration 1 gene (RCJMB04_8m13)	419723	Transcription factor	Vasculogenesis and Hematopoiesis	Endothelial and erythroid cell precursors
**+1.96**	HHEX	Hematopoietically expressed homeobox	396182	Transcription factor	Vasculogenesis and Hematopoiesis	Blood islands and anterior intestinal portal
**-1.71**	SOX17	SRY (sex determining region Y)-box 17	428534	Transcription factor	Endoderm differentiation	Definitive endoderm
**-2.73**	CDH2	Cadherin 2, type 1, N-cadherin (neuronal)	414745	Cell adhesion molecule	Epithelial cell adhesion	Primitive streak and neurectoderm
**-3.39**	CXCL14	Chemokine (C-X-C motif) ligand 14	395451	Chemokine	Directional cellular migration	Ectodermal derivatives
**-3.4**	CDH1	Cadherin 1, type 1, E-cadherin (epithelial)	415860	Cell adhesion molecule	Epithelial cell adhesion	Ectoderm
**-4.52**	WNT8A	Wingless-type MMTV integration site family, member 8A	396543	Secreted factor	Mesoderm and neurectoderm patterning	Primitive streak and paraxial mesoderm
**-4.58**	DLL1	Delta-like 1	395820	Transmembrane protein	Left-right asymmetry determination	Primitive streak and paraxial mesoderm
**-5.48**	FOXC2	Forkhead box C2 (MFH-1, mesenchyme forkhead 1)	396039	Transcription factor	Lymphatic development	Cephalic and paraxial mesoderm
**-8.24**	CYP26C1	Cytochrome P450, family 26, subfamily C, polypeptide 1	423806	Catabolizing enzyme	Retinoic acid degradation	Anterior mesoderm and neurectoderm
**-8.32**	FGF18	Fibroblast growth factor 18	395453	Secreted factor	Chondrogenesis and osteogenesis	Primitive streak and rostral neurectoderm
**-8.82**	FGF19	Fibroblast growth factor 19	395394	Secreted factor	Brain and eye development	Primitive streak and head mesenchyme
**-9.75**	DKK1	Dickkopf homolog 1	374156	Secreted WNT inhibitor	Head induction	Axial mesendoderm and Hensen's node
**-9.84**	LOC421238	Hypothetical gene supported by CR405884	421390	-	-	-
**-10.15**	CXCR4	Chemokine (C-X-C motif) receptor 4	395324	Chemokine receptor	Cardiogenesis	Definitive mesendoderm
**-12.25**	FZD8	Frizzled homolog 8	374059	Transmembrane receptor	Anterior brain patterning	Anterior neurectoderm
**-12.92**	MSGN1	Mesogenin 1	395419	Transcription factor	Somitogenesis	Paraxial mesoderm
**-15.29**	OTX2	Orthodenticle homeobox 2	395191	Homeobox gene	Specification of rostral neurectoderm	Neuroectoderm
**-16.93**	Crescent	Crescent gene (LOC395991 locus)	395991	Secreted WNT inhibitor	Cardiogenesis	Prechordal plate and anterior endoderm
**-24.45**	FST	Follistatin	396119	Secreted activin inhibitor	Stem cell renewal (?)	Neural plate and paraxial mesoderm

Listed genes exhibit greater than 1.7-fold change (lower bound) in expression of Hb-eGFP+ *versus* Hb-eGFP- cells. Gene function and expression patterns are given where known and were obtained from GEISHA (http://geisha.arizona.edu/geisha) and from the literature.
